# Ascorbate protects human kidney organoids from damage induced by cell-free hemoglobin

**DOI:** 10.1242/dmm.050342

**Published:** 2023-12-01

**Authors:** Julie Bejoy, Justin M. Farry, Eddie S. Qian, Curtis H. Dearing, Lorraine B. Ware, Julie A. Bastarache, Lauren E. Woodard

**Affiliations:** ^1^ Department of Medicine, Division of Nephrology and Hypertension, Vanderbilt University Medical Center, Nashville, TN 37232, USA; ^2^ Department of Biomedical Engineering, Vanderbilt University, Nashville, TN 37232, USA; ^3^ Vanderbilt Experimental Research Training Inclusion Community Engagement Skills (VERTICES) program, Vanderbilt University, Nashville, TN 37232, USA; ^4^ Department of Medicine, Division of Allergy, Pulmonary and Critical Care Medicine, Vanderbilt University Medical Center, Nashville, TN 37232, USA; ^5^ Department of Pathology, Microbiology and Immunology, Vanderbilt University Medical Center, Nashville, TN 37232, USA; ^6^ Department of Cell Biology, Vanderbilt University Medical Center, Nashville, TN 37232, USA; ^7^ U.S. Department of Veterans Affairs, Nashville, TN 37212, USA

**Keywords:** Acute kidney injury, Cell-free hemoglobin, Induced pluripotent stem cells, Kidney, Organoids, Sepsis

## Abstract

Sepsis-associated acute kidney injury is associated with high morbidity and mortality in critically ill patients. Cell-free hemoglobin (CFH) is released into the circulation of patients with severe sepsis and the levels of CFH are independently associated with mortality. CFH treatment increased cytotoxicity in the human tubular epithelial cell line HK-2. To better model the intact kidney, we cultured human kidney organoids derived from induced pluripotent stem cells. We treated human kidney organoids grown using both three-dimensional and transwell protocols with CFH for 48 h. We found evidence for increased tubular toxicity, oxidative stress, mitochondrial fragmentation, endothelial cell injury and injury-associated transcripts compared to those of the untreated control group. To evaluate the protective effect of clinically available small molecules, we co-treated CFH-injured organoids with ascorbate (vitamin C) or acetaminophen for 48 h. We found significantly decreased toxicity, preservation of endothelial cells and reduced mitochondrial fragmentation in the group receiving ascorbate following CFH treatment. This study provides direct evidence that ascorbate or ascorbic acid protects human kidney cells from CFH-induced damage such as that in sepsis-associated acute kidney injury.

## Teaser

**Summary:** Cell-free hemoglobin (CFH) treatment of human kidney organoids serves as an *in vitro* model of sepsis-associated acute kidney injury. Treatment with ascorbate mitigated CFH-induced injury of kidney cells.

## INTRODUCTION

Sepsis has an annual incidence in the USA of more than 750,000 cases per year (Hajj et al., 2018). The cost of care in the hospital is $24.3 billion, with mortality rates of 25-30% and high rates of long-term disability in people who recovered from sepsis (Lagu et al., 2012; Martin et al., 2003). There are currently no specific therapies for sepsis or sepsis-associated acute kidney injury (AKI) other than antimicrobial drugs, dialysis and fluids. To model sepsis-associated AKI in animals, lipopolysaccharide (LPS), a component of the cell wall of Gram-negative bacteria, has been widely used. However, using LPS to model sepsis-associated AKI *in vitro* has produced controversial results (Langenberg et al., 2014; Peerapornratana et al., 2019; Zhang et al., 2021). Herein, we investigated another key contributor of kidney injury in sepsis, namely, hemoproteins (Boutaud et al., 2010; Moore et al., 1998). Circulating cell-free hemoglobin (CFH) is not detectable in healthy humans. But plasma CFH levels are elevated in 80% of patients with sepsis; this elevation is sustained over time and strongly and independently associated with mortality (Adamzik et al., 2012; Janz et al., 2013). Cytokine signaling during septic inflammation causes eryptosis, which releases CFH into circulation. The CFH released can injure the kidneys through tubular obstruction, tubular epithelial injury, endothelial dysfunction, the production of reactive oxygen species (ROS) and pro-inflammatory signaling (Kerchberger and Ware, 2020).

Human-specific aspects of AKI and nephrotoxicity have been elucidated by modeling in human renal cells that are cultured and injured *ex vivo*. Existing tissue culture models of AKI apply nephrotoxic drugs that cause toxicity and cell death to kidney cells (Bejoy et al., 2022; Lawrence et al., 2022; Morizane and Bonventre, 2017). A recent study showed that treatment of human kidney organoids with hemin, an iron-containing porphyrin released from red blood cells during hemolysis, caused oxidative damage, expression of injury markers and fibrosis, which was reduced via treatment with a histone deacetylase inhibitor (Przepiorski et al., 2022). Our prior studies on sepsis-associated AKI found that CFH treatment of an immortalized proximal tubular epithelial cell line (HK-2) increased cytotoxicity, suggesting that CFH can directly injure the renal tubular epithelium (Shaver et al., 2019). Although immortalized tubule cell lines such as HK-2 can be injured by treatment with CFH, they are monolayers grown on stiff plastic that lack connected mitochondrial networks. Human kidney organoid models of AKI permit modeling of interactions between kidney cell types to better understand the kidney response to nephrotoxicants (Bejoy et al., 2022; Gupta et al., 2022; Lawrence et al., 2022; Morizane and Bonventre, 2017). In this study, we generated a tissue culture model of CFH-induced AKI in human kidney organoids and measured differences in cell viability and cytotoxicity.

We chose to evaluate two small molecules, acetaminophen (APAP) and ascorbic acid (AA, also known as ascorbate or vitamin C), for protection from the toxic and oxidative effects of CFH on human kidney organoids. When administered at safe, clinically relevant doses, APAP can reduce the Fe^4+^ in ferryl-CFH to the less reactive Fe^3+^ form (Boutaud et al., 2010; Boutaud and Roberts, 2011; González-Sánchez et al., 2009). The clinical importance of this effect is highlighted by data from our phase IIa randomized clinical trial indicating that treatment of patients with sepsis who had detectable levels of plasma CFH with APAP was safe, decreased oxidative injury as measured by plasma F2-isoprostanes, and improved renal function (Janz et al., 2015). The other small molecule tested was AA, a water-soluble essential nutrient and antioxidant involved in tissue repair, including collagen remodeling. Decreased plasma levels of AA have been reported in patients with sepsis (Voigt et al., 2002). Supplementation of AA had protective effects on endothelial permeability in CFH-treated endothelial cells (Kuck et al., 2018). Clinical trial data on the use of AA in sepsis have produced inconsistent results, with some studies finding a benefit while others do not, resulting in some controversy in the field (Zhu et al., 2022). The need for an *in vitro*, isolated human system in which to study both CFH-induced renal cell damage as well as APAP and/or AA protection led to development of our kidney organoid models of CFH-induced injury.

## RESULTS

### CFH treatment of human kidney organoids grown on transwells increases cell death and ROS

In a prior study, we found that CFH increased cytotoxicity in renal tubular cells (HK-2) (Shaver et al., 2019). We examined viability by 3-(4,5-dimethylthiazol-2-yl)-2,5-diphenyltetrazolium bromide (MTT) assay and found that the CFH-treated group had a substantial decline in viability (*P*=0.0001; Fig. S1Ai). We also looked at cytotoxicity and found higher lactate dehydrogenase (LDH) release in the CFH-treated groups (*P*<0.005; Fig. S1Aii). Next, we stained the CFH-treated cells with apoptosis and proliferation markers to understand the mechanism of cytotoxicity. Proliferation as evidenced by Ki-67 staining was not different between the groups (Fig. S1C). However, cleaved caspase-3 staining was increased in the CFH-treated group (Fig. S1Bi,ii). These results suggest that CFH can induce cytotoxicity in tubule cells via induction of apoptosis. As heme redox cycling between the ferric and ferryl states generates globin radicals that cause lipid peroxidation (Reeder and Wilson, 2005), we measured levels of peroxidation in CFH-treated HK-2 cells. CFH treatment increased the concentration of the lipid peroxidation product malondialdehyde (MDA) from ∼3.5 µM to 22.5 µM (*P*<0.0001; Fig. S1D). However, multiple MTT viability assay trials produced variable results (Fig. S1E), suggesting the need for more advanced models with better differentiated tubular epithelium and/or other cell types in order to recapitulate kidney injury *in vitro*.

**Fig. 1.**
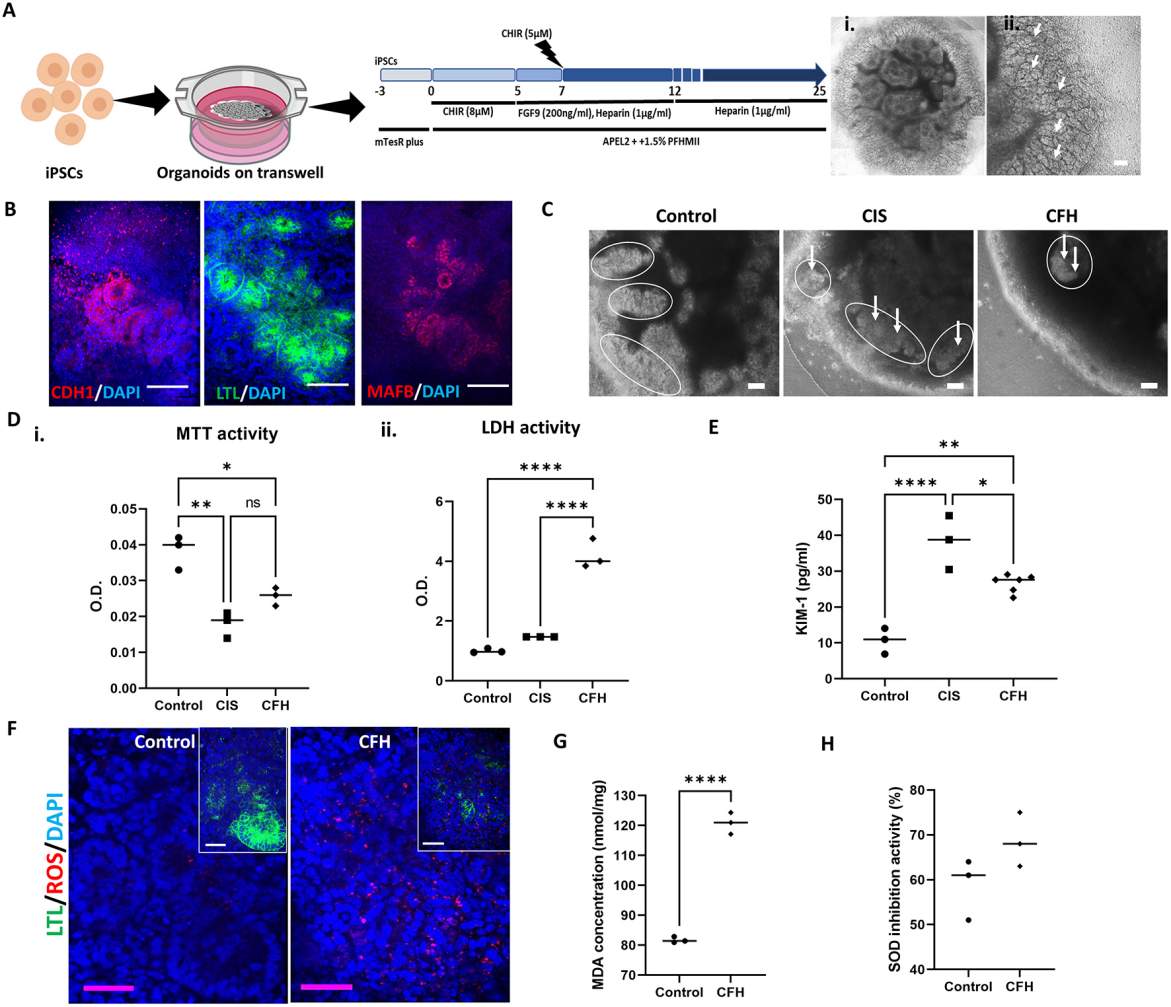
**CFH increases cytotoxicity and reduces viability of cells within transwell kidney organoids via ROS.** (A) Schematic outline of the protocol for differentiating iPSCs into kidney organoids on transwell dishes and a brightfield image of the whole kidney organoids at day 25 (i), with a high-magnification image showing S-shaped bodies (arrows) (ii). Scale bar: 50 µm (ii). Created with BioRender.com and PowerPoint. (B) Immunostaining of the resulting organoid shows positive staining for markers of the distal tubule (CDH1), proximal tubule (LTL), and podocytes (MAFB) (*n*=10 organoids from more than three independent experiments). Scale bars: 100 µm. (C) The brightfield images of the whole organoids treated with either cisplatin (CIS) or cell-free hemoglobin (CFH) with lost tubule segments (white arrows); the outlined regions represent the differentiated areas (*n*=3 organoids from three independent experiments). Scale bars: 50 µm. (D) The impact of CFH on the viability as measured by MTT activity (i) and cytotoxicity as measured by LDH activity (ii) of cells within kidney organoids (*n*=5 independent experiments; data shown are from one experiment). O.D., optical density. (E) Quantification of the amount of KIM-1 released into the medium for the CFH-treated organoids compared to that for the control group as measured by ELISA. (F) Co-staining for ROS (red), DAPI to mark nuclei (blue) and the proximal tubule marker LTL (green) in human kidney organoids (*n*=3 organoids from three independent experiments). Scale bars: 50 µm. (G) Malondialdehyde (MDA) assay in untreated organoids versus kidney organoids receiving the CFH treatment. (H) Superoxide dismutase (SOD) assay for the control and CFH-treated organoid groups (*n*=3). Bars show median values. ns, not significant; *P<0.05; **P<0.01; ****P≤0.0001 (one-way ANOVA with Tukey's multiple comparisons for D,E, unpaired two tailed *t*-test for G,H).

Therefore, we used human induced pluripotent stem cell (iPSC)-derived kidney organoids, which include multiple cell types. We tested organoids derived using the transwell-based method (Takasato et al., 2015) (Fig. 1A). The process involves generating intermediate mesoderm from primitive streak with the Wnt activator CHIR99021 (hereafter CHIR), followed by induction of nephrogenesis with FGF9. During nephrogenesis, cells grown in a monolayer were harvested and transferred onto the transwells as a cell pellet to create an air-liquid interface for the rest of the culture time until day 25. Brightfield images of the day 25 organoids revealed S-shaped bodies and renal vesicles (Fig. 1A). Immunostaining analysis of the mature organoids confirmed the presence of distal tubule cells (marked by E-cadherin or CDH1), proximal tubule cells (marked by lotus tetragonolobus lectin or LTL) and podocytes (marked by MAF BZIP transcription factor B or MAFB) (Fig. 1B) (Bejoy et al., 2022). The efficiency of differentiation was 60-70% as determined by whole-mount staining. We used a LDH release assay to determine the concentration of CFH (1 mg/ml) that caused significant toxicity to the organoids (Fig. S2A). After 48 h of treatment with CFH (1 mg/ml) or the positive control cisplatin (CIS; 5 µM), the organoids on transwells were examined for toxicity and viability. Tubule segments and S-shaped bodies lost integrity following CFH treatment (Fig. 1C). We observed a modest increase in kidney injury molecule 1 (KIM-1, also known as HAVCR1 and TIM-1) release when the cells were treated with both cisplatin and CFH compared to that in the CFH-only treated group, but this was not significant (Fig. S2B). The tubule segments were disrupted in the group receiving either the nephrotoxic drug cisplatin or CFH, showing a comparable injury (Fig. 1C; Fig. S2C). Viability was also significantly reduced after CFH treatment (*P*<0.001; Fig. 1Di). The amount of cytotoxicity was higher in the CFH group than either the negative control group (*P*<0.0001) or the cisplatin-treated positive control group (*P*<0.0001; Fig. 1Dii). Exposure to CFH increased the amount of KIM-1 released into the cell culture medium as measured by enzyme-linked immunosorbent assay (ELISA) (*P*<0.001; Fig. 1E). Oxidative stress is one of the key pathways by which increased levels of circulating CFH can cause kidney damage. Therefore, we indirectly measured production of ROS in response to CFH (Fig. 1F). CFH treatment increased the fluorescence detection of CellROX, a deep red probe used to measure cellular oxidative stress. CellROX was detected in both the LTL-positive tubules and LTL-negative areas, suggestive of increased ROS present throughout the CFH-treated kidney organoids (Fig. 1F). Similar results have been reported elsewhere (Agyemang et al., 2021). To understand the downstream effects of the increased oxidative stress, we measured the lipid peroxidation product MDA and found that the CFH group had a higher MDA concentration (120 nmol/mg) than that of the control group (80 nmol/mg; *P*<0.0001; Fig. 1G). Superoxide dismutase (SOD) converts superoxide to hydrogen peroxide in the presence of increased ROS. Glutathione peroxidase or catalase subsequently removes the hydrogen peroxide. SODs thereby limit the development of extremely aggressive ROS and lessen the damage (Afonso et al., 2007). Therefore, we measured SOD activity after CFH treatment. In the CFH-treated group, there was an increase in SOD activity (Fig. 1H) albeit non-significant, which indicates the presence of free radicals.

### CFH induces cytotoxicity, reduces viability and increases oxidative stress in 3D human kidney organoids

**Fig. 2.**
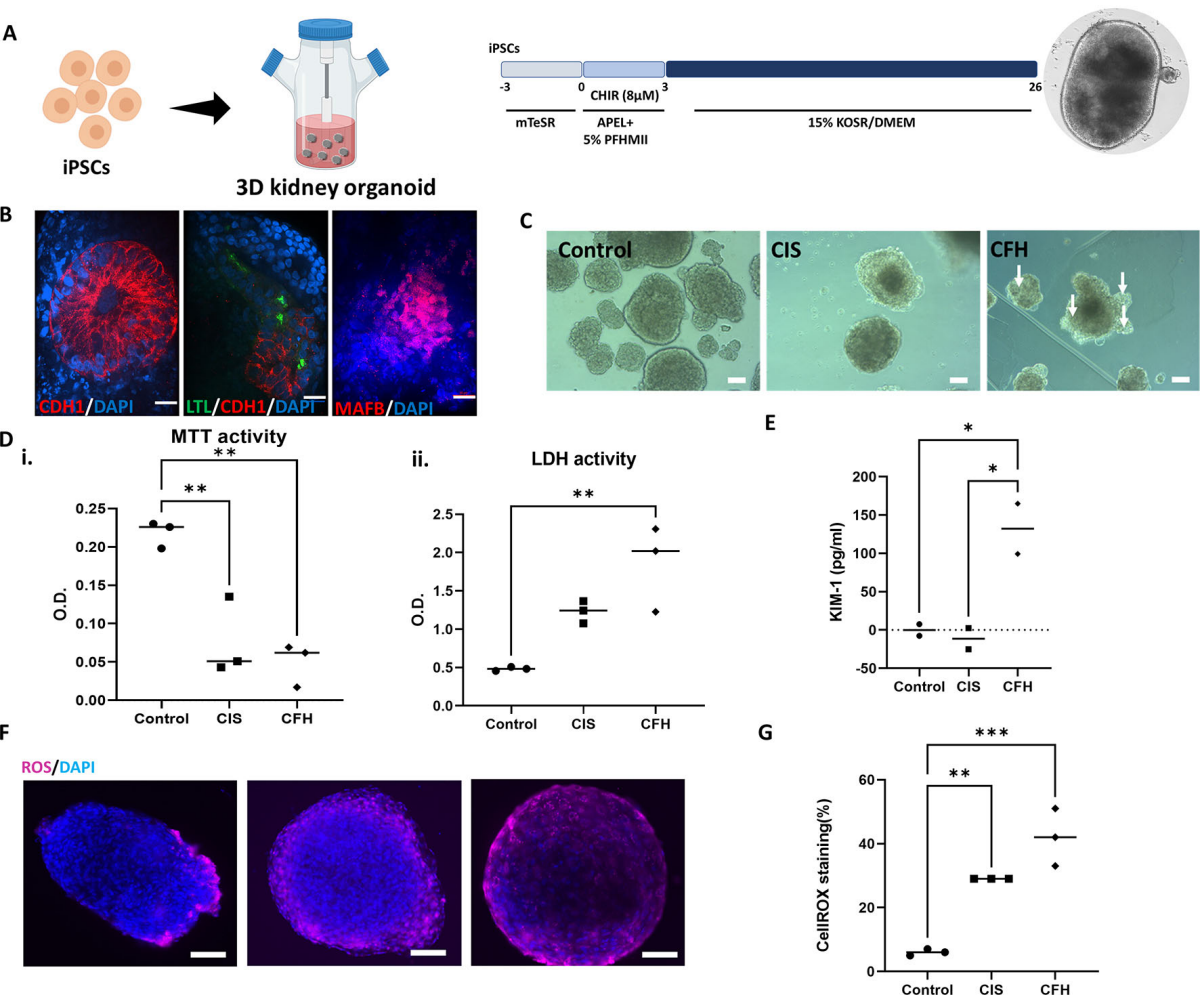
**CFH increases cytotoxicity and reduces viability of cells within 3D human kidney organoids via ROS.** (A) Schematic of human kidney organoid differentiation growth according to the 3D bioreactor protocol. Created with BioRender.com and PowerPoint. (B) The 3D kidney organoids stained for markers of various nephron segments including the distal tubule (CDH1, red), proximal tubule (LTL, green) and glomerular podocytes (MAFB, red); nuclei are stained with DAPI (blue). Scale bars: 20 µm (*n*=10 organoids from more than three independent experiments). (C) Brightfield images of organoids treated with CFH and the positive control, the nephrotoxicant cisplatin (CIS) (*n*=3 organoids from three independent experiments). Arrows indicate injured organoids losing the integrity of cell membrane. Scale bars: 100 µm. (D) Cell viability as measured by MTT activity (i) and cytotoxicity as measured by LDH activity (ii) in the 3D human kidney organoids (*n*=3). (E) ELISA for KIM-1 release in the medium. (F) Intracellular ROS production was measured with CellROX fluorescence to indicate ROS levels (red); nuclei were stained with DAPI (blue). Scale bars: 100 µm. (G) Quantification of CellROX. Bars show median values. *P<0.05; **P<0.01; ***P<0.001 (one-way ANOVA with Tukey's multiple comparisons for D,E,G).

To confirm these results, we employed a bioreactor-based strategy to create bioreactor-grown suspension three-dimensional (3D) organoids as described by Przepiorski et al. (2018) (Fig. 2). After 3 days of embryoic body development in the presence of CHIR, the cells were treated with medium containing Knockout Serum Replacement^TM^ (KOSR; Fig. 2A). During the late stages of differentiation, the organoids were grown in spinner-flask bioreactors and harvested on day 25 (Fig. 2A). We stained the organoids to detect the presence of distal tubule cells by CDH1 staining, proximal tubule cells with LTL staining and glomeruli with MAFB staining (Fig. 2B). The average efficiency of differentiation as calculated by staining was between 20 and 25%, which was lower than that of the transwell-based method. A decrease in the structural integrity of the organoids after treatment with CFH could be seen by brightfield imaging (Fig. 2C). Suspension organoids, similar to transwell organoids, demonstrated lower viability and increased toxicity (Fig. 2Di,ii). Release of KIM-1 into the cell culture medium was also increased by CFH (*P*<0.05; Fig. 2E). We measured generation of intracellular ROS via image quantification of CellROX fluorescence and found the levels to be substantially higher in CFH-treated organoids (40%) than in cisplatin-treated cells or control cells (Fig. 2F,G). In conclusion, oxidative stress is a likely contributor to CFH-induced cellular apoptosis, cytotoxicity and decreased viability in kidney cells. As only 20-25% of each 3D kidney organoid stained positively for markers of the nephron, the toxicity could possibly be due to off-target cell types. However, the presence of KIM-1, a highly specific marker of proximal tubular injury, suggests that at least a portion of the cytotoxicity was directly attributable to CFH-induced proximal tubular injury.

### CFH increases endothelial cell depletion, tubule cell damage, cytokines and renal fibrosis

**Fig. 3.**
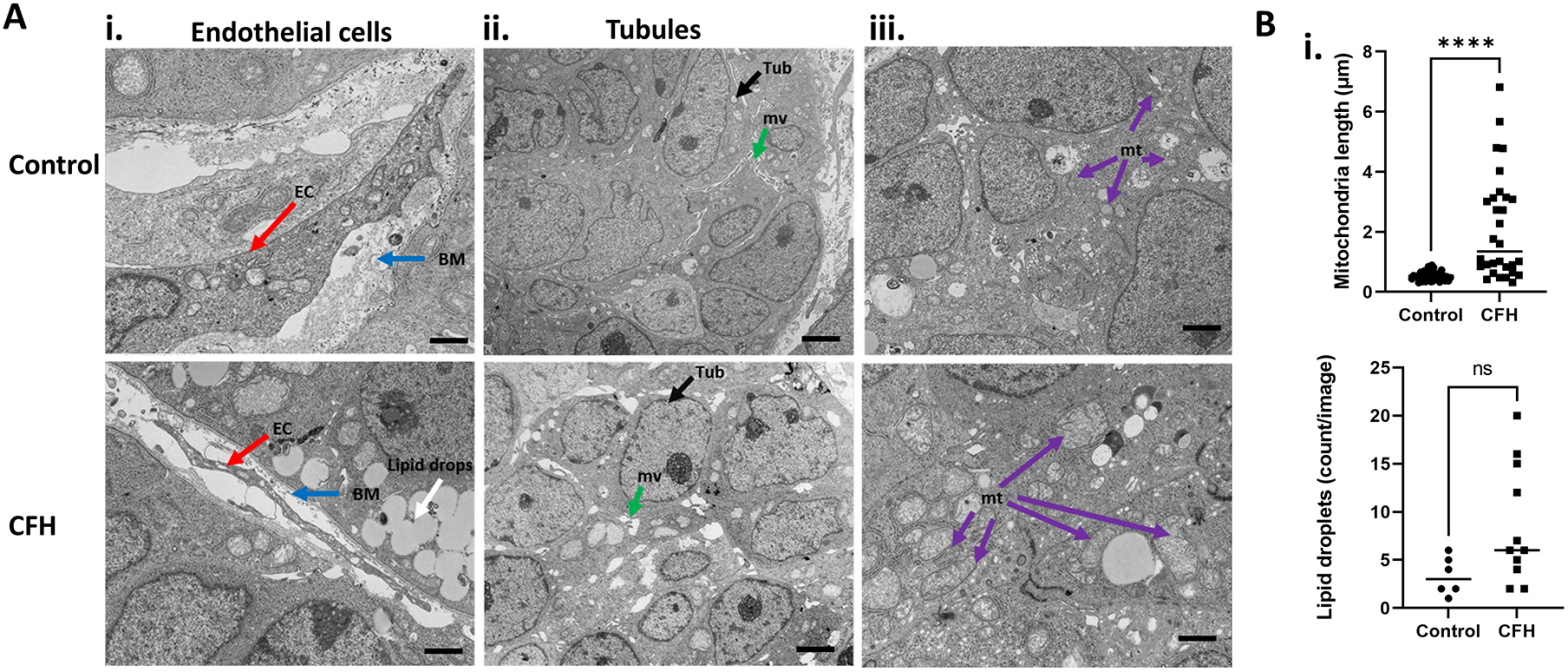
**Kidney organoids treated with CFH have endothelial cell atrophy, loss of tubular cell-cell junctions and swollen mitochondria.** (A) Representative transmission electron microscopy images of endothelial cells (i), tubules (ii) and mitochondria (iii) in human kidney organoids treated with or without CFH. Endothelial-like cells (ECs; red arrows) near the basement membrane (BM, blue arrows), lipid drops (white arrow) and tubule-like cells (black arrows) with microvilli (mv, green arrows) and mitochondria (purple arrows) are indicated. Images are representative of two organoids from one experiment. Scale bars: 1 µm. (B) Quantification of mitochondrial length (i) and lipid droplets (ii) in the CFH-treated organoids compared to those in control. Bars show median values. ns, not significant; ****P≤0.0001 (unpaired two-tailed *t*-test).

We performed transmission electron microscopy (TEM) analyses and found that fewer endothelial cells were present in human kidney organoids that were treated with CFH (Fig. 3A) compared to those in untreated transwell organoids. The CFH-treated organoids had more apoptotic cells and an increased amount of cell debris present (Fig. S3). Additionally, we noted that the ability of tubule cells to form microvilli was diminished in the treated organoids compared with that in controls (Fig. 3Aii). We also observed mitochondrial swelling in the CFH-treated group, which is commonly associated with cell death (*P*<0.0001; Fig. 3Aiii,Bi). CFH treatment also increased the frequency of lipid droplets in the organoids (Fig. 3A,Bii).

**Fig. 4.**
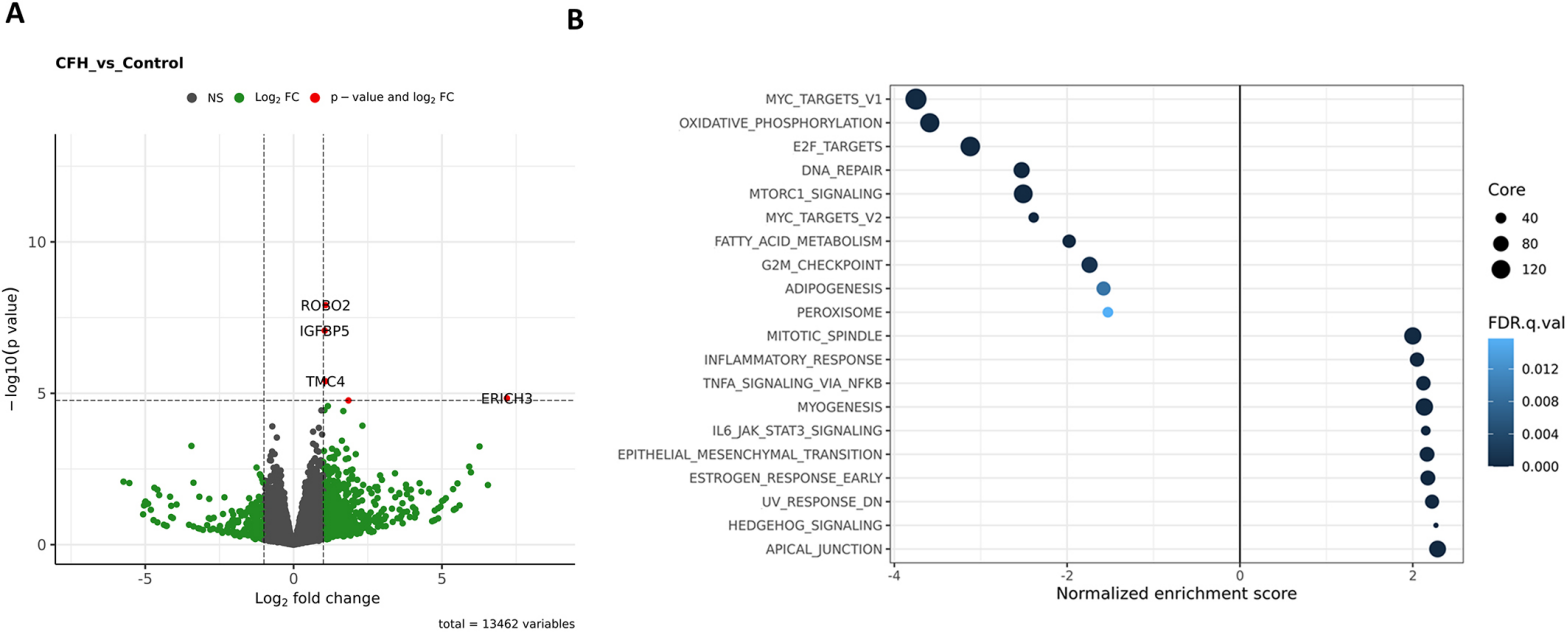
**Bulk RNA sequencing reveals increase in cytokine- and fibrosis-related genes as well as upregulation of inflammatory pathways in CFH-treated organoids.** (A) Volcano plot showing the differential expression of genes associated with injury in CFH-treated organoids compared to that in control. The right side of the plot indicates upregulated transcripts and the left side shows downregulated transcripts in the CFH group compared to the control group. Green dots indicate the statistically significantly regulated genes that had a fold change of 2 (positive or negative) with *P*-value <0.05. Red dots indicate the genes with the greatest fold change that had log_10_*P*≥5 and log_2_(fold change or FC)>2. *ROBO2*, *IGFBP5*, *TMC4* and *ERICH3* were the top enriched genes. NS, not significant (gray dots). (B) Top enriched gene sets in each cluster with functions in biological processes showing the upregulation of genes involved in pathways including inflammatory response, TNFα signaling, IL6-JAK-STAT3 signaling, as well as the epithelial to mesenchymal transition in the CFH group. *n*=3 samples per group.

Next, we compared CFH-treated and untreated transwell organoids by RNA sequencing (Fig. 4). Four genes were highly enriched in CFH-treated groups compared to control groups: glutamate-rich 3 (*ERICH3*), roundabout guidance receptor 2 (*ROBO2*), insulin-like growth factor-binding protein 5 (*IGFBP5*) and transmembrane channel-like 4 (*TMC4*) (Fig. 4A). About 26 genes were expressed with log_2_(fold change or FC) more than 5 in the CFH group compared to the control groups (Table S2). Genes regulating tubule development including lipoprotein-related protein 2 (*LRP2*) (log_2_FC=2.139797454) and cubilin (*CUBN*) (log_2_FC=2.55186658) were significantly increased in the CFH group, suggesting differentiation under injury (Fig. 4A). We discovered a twofold rise in the transcript levels of the AKI injury biomarker *KIM-1* (log_2_FC=2.09445508). Additionally, we discovered that cytokines associated with acute or chronic kidney injury, C-X-C motif chemokine ligands 1, 6, 8 and 10 (*CXCL1*, *CXCL6*, *CXCL8* and *CXCL10*) had increased transcript abundance in organoids receiving the CFH treatment (Fig. 4A). Renal fibrosis-associated transcripts (Liu et al., 2017), including matrix metalloproteinase-1 (*MMP1*), matrix metalloproteinase-7 (*MMP7*), matrix metalloproteinase-9 (*MMP9*), serpin family E member 1 (*SERPINE1*) and many collagens (*COL6A5*, *COL6A6*, *COL7A1*, *COL8A1*, *COL15A1*, *COL19A1* and *COL21A1*), were also elevated in the CFH-treated organoid group (Table S2). When analyzed for top enriched gene sets, CFH-treated organoids had enrichment in ‘inflammatory response’, ‘TNFα signaling’, ‘myogenesis’, ‘IL6-JAK-STAT3 signaling’, ‘hedgehog signaling’ and ‘epithelial to mesenchymal transition’ (Fig. 4B).

### AA reduces the cytotoxicity triggered by CFH in kidney organoids via reduction of ROS

**Fig. 5.**
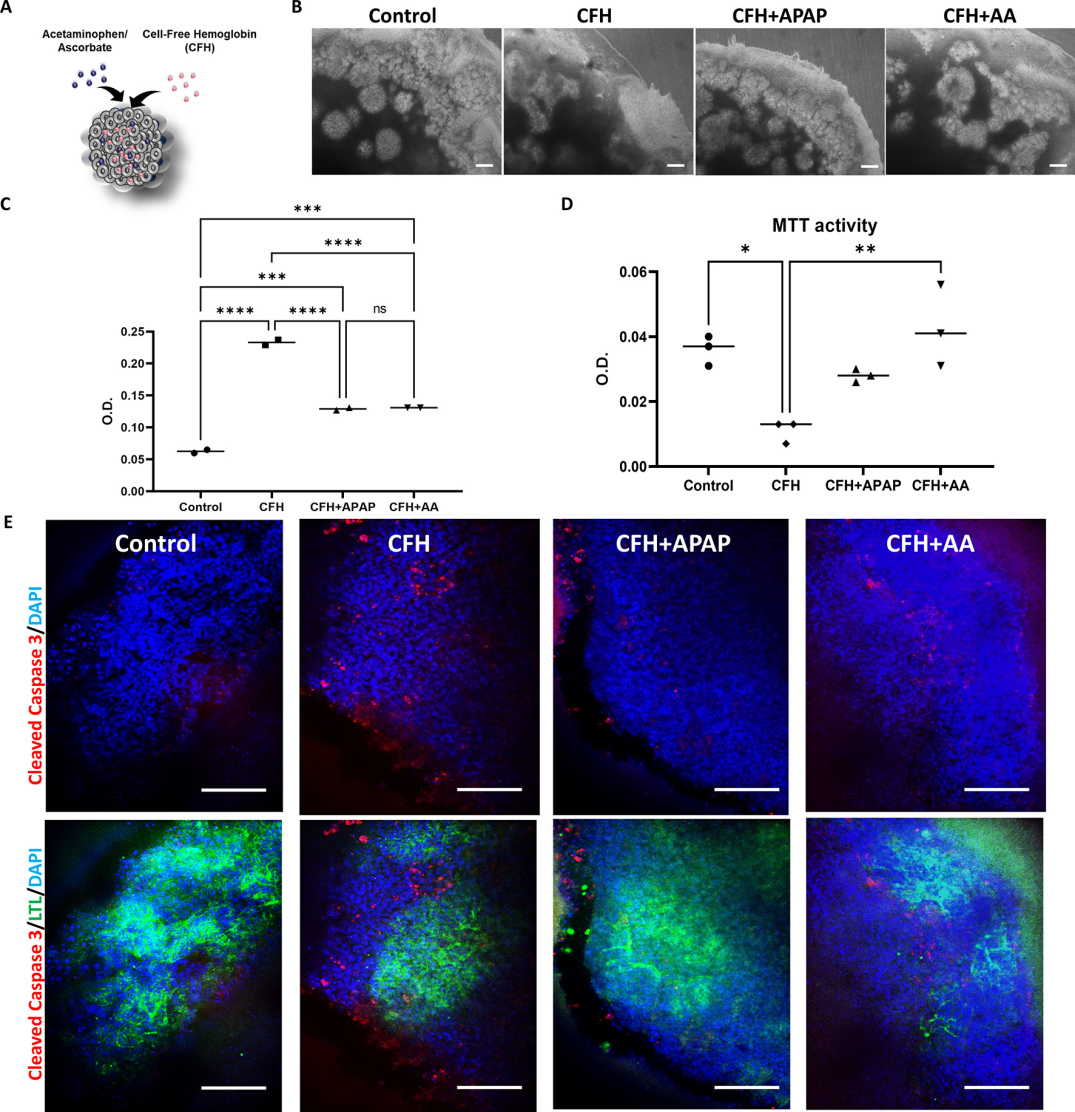
**Ascorbate improves viability and reduces apoptosis triggered by CFH treatment in kidney organoids.** (A) Transwell organoids were treated at day 19 for 48 h with either CFH (1 mg/ml), CFH and acetaminophen (APAP; 1000 nM), or CFH and ascorbate (AA; 200 nM). (B) Brightfield images of the corresponding organoids show preserved nephron segments in white and dying areas of the organoids in black. Scale bars: 50 µm. (*n*=10 organoids from ten independent experiments) (C) Co-treatment with either APAP or AA analyzed for toxicity using LDH assay at 48 h (*n*=3 organoids from three independent experiments). (D) Viability was determined by MTT assay in the co-treated groups at 48 h in comparison to that of control and CFH-alone groups (*n*=3 organoids from three independent experiments). (E) Human kidney organoids were untreated (control), treated with CFH (CFH), treated with CFH and APAP (CFH+APAP), or treated with CFH and AA (CFH+AA) and co-stained for cleaved caspase-3 (red, stains cells undergoing apoptosis), the proximal tubule marker LTL (green) and nuclei with DAPI (blue). Scale bars: 100 µm (*n*=3 organoids from three independent experiments). Bars show median values. ns, not significant; *P<0.05; **P<0.01; ***P<0.001; ****P≤0.0001 (one-way ANOVA with Tukey's multiple comparisons for C,D).

We co-treated transwell kidney organoids for 48 h with CFH and either APAP or AA (Fig. 5A). In brightfield images, more tubule segments were intact in organoids that were co-treated with APAP or AA than in organoids treated with CFH alone (Fig. 5B). The CFH+AA group outperformed the CFH+APAP group in terms of tubular segment preservation (Fig. 5B). We measured cytotoxicity by LDH assay and found that both APAP and AA reduced toxicity in the organoids within the first 24 h (Fig. 5C). We found that AA treatment had enhanced viability compared to that upon APAP treatment (*P*<0.01; Fig. 5D). KIM-1 ELISA analysis indicated reduced KIM-1 release in the APAP group but it was not statistically significant (Fig. S4). We also investigated whether there was a reduction in apoptosis as indicated by cleaved caspase-3 staining. Although the APAP group did not show any reduction in staining, the AA group did show reduced cleaved caspase-3 staining (Fig. 5E). Next, we examined the positive effects of these compounds on ROS, as we discovered that CFH promotes cytotoxicity in kidney organoids via ROS. Both compounds reduced ROS levels in the organoids (Fig. S5A). APAP treatment slightly reduced lipid peroxidation (Fig. S5B), but there was no change in SOD activity in any treatment group (Fig. S5C).

### CFH treatment of human kidney organoids causes cytokine release that is reduced by co-treatment with AA

**Fig. 6.**
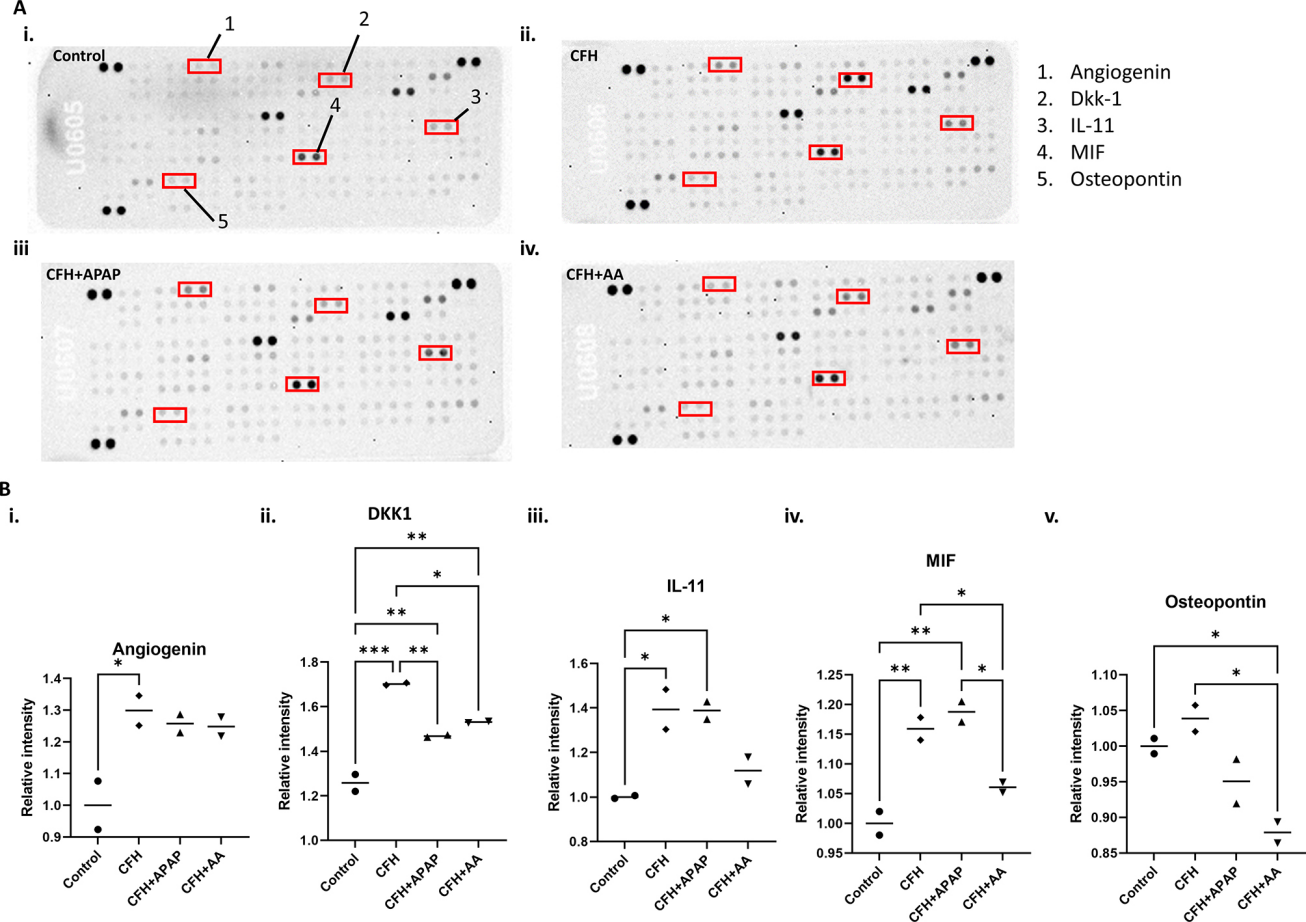
**CFH treatment induces cytokine release that was altered after co-treatment with APAP and AA.** (A) Cytokine array analysis of culture media collected from dishes containing human kidney organoids from the following 48 h treatment groups: (i) untreated control, (ii) CFH, (iii) CFH and APAP (CFH+APAP), and (iv) CFH and AA (CFH+AA). Factors that appeared to change between groups are marked in the red boxes. (B) Quantification of cytokine levels in ImageJ software for: (i) angiogenin, (ii) DKK1, (iii) IL-11, (iv) MIF and (v) osteopontin. Bars show median values. *P<0.05; **P<0.01; ***P<0.001 (one-way ANOVA with Tukey's multiple comparisons).

We next examined the cytokines produced in the medium in response to CFH using the Proteome Profiler Human XL Cytokine Array (Fig. 6A). Five different cytokines were expressed differentially in the CFH group compared to the control group: DKK1, angiogenin (ANG), IL-11, osteopontin (SPP1) and macrophage migration inhibitory factor (MIF). Three additional cytokines, NGAL (also known as LCN2), SDF1α (CXCL12) and FGF-19, had a trend toward being increased in the CFH group compared to the control group (Fig. S6). Cells release the majority of these cytokines in response to inflammation and damage. Co-treatment with APAP did not significantly change the expression of any of the cytokines. However, there was a trend toward reduction in the levels of NGAL and FGF-19 (Fig. S6). Treatment with AA resulted in a trend toward reduction of angiogenin, IL-11 and NGAL, with significant differences in the levels of DKK1, MIF and osteopontin (Fig. 6; Fig. S6C).

### AA reduces the mitochondrial damage induced by CFH in kidney organoids

**Fig. 7.**
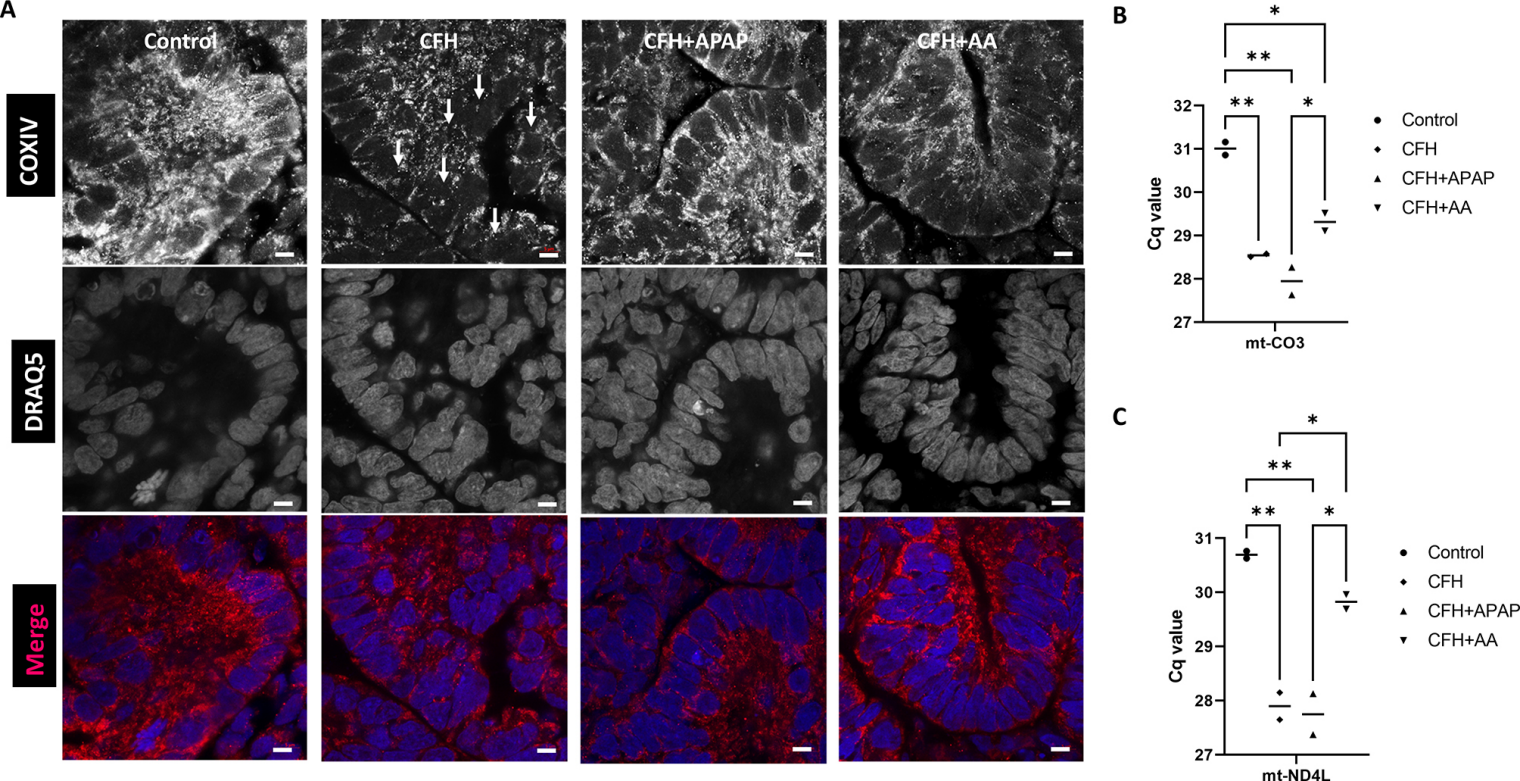
**AA reduces mitochondrial damage in CFH-treated human kidney organoids.** (A) Immunostaining with mitochondrial marker COX-IV (red) showing segmented mitochondria (white arrows) within a tubule segment of CFH-treated transwell organoids co-stained with DRAQ5 to mark the nuclei (blue; *n*=3 organoids from three independent experiments). Scale bars: 5 μm. (B,C) The levels of mitochondrial DNA released to the cell culture medium due to segmentation were evaluated with real-time quantitative PCR analysis of (B) *MT-CO3* and (C) *MT-ND4L*. Bars show median values. *P<0.05; **P<0.01 (one-way ANOVA with Tukey's multiple comparisons for B,C).

To assess CFH-induced mitochondrial segmentation in the renal proximal tubular epithelium, we used high-magnification confocal imaging of mitochondria in the treated organoids (Fig. 7A). After CFH treatment, we stained for the mitochondrial marker COX-IV and found damaged mitochondrial segments throughout each cell. Compared to the CFH-alone group, both APAP and AA co-treatments improved the structure of the mitochondrial organelle network within the organoids (Fig. 7A). The co-treated groups also maintained LTL expression (Fig. S7). Next, we performed real-time PCR (RT-PCR) analysis of mitochondrial DNA (mtDNA) in the cell culture medium to quantify the degree of mitochondrial damage and subsequent mtDNA release into the medium after CFH treatment (Fig. 7B,C). Quantification cycle (C_q_) values for the mitochondrial genes *MT-CO3* and *MT-ND4L* were significantly lower in the CFH-treated group than in the untreated group (*P*<0.01), indicating that higher levels of mtDNA were released into the organoid medium in the presence of CFH. However, the C_q_ values for the CFH+APAP group were similar to those in the CFH-alone group, so APAP did not reduce mtDNA release (Fig. 7B,C). The CFH+AA group, however, had a C_q_ value approaching that of the untreated control group, indicating that mtDNA release into the medium was mitigated by AA (Fig. 7B,C). Overall, we found that that AA lowered mitochondrial segmentation caused by CFH treatment.

### AA maintains CDH5 expression within the CFH-treated organoids

**Fig. 8.**
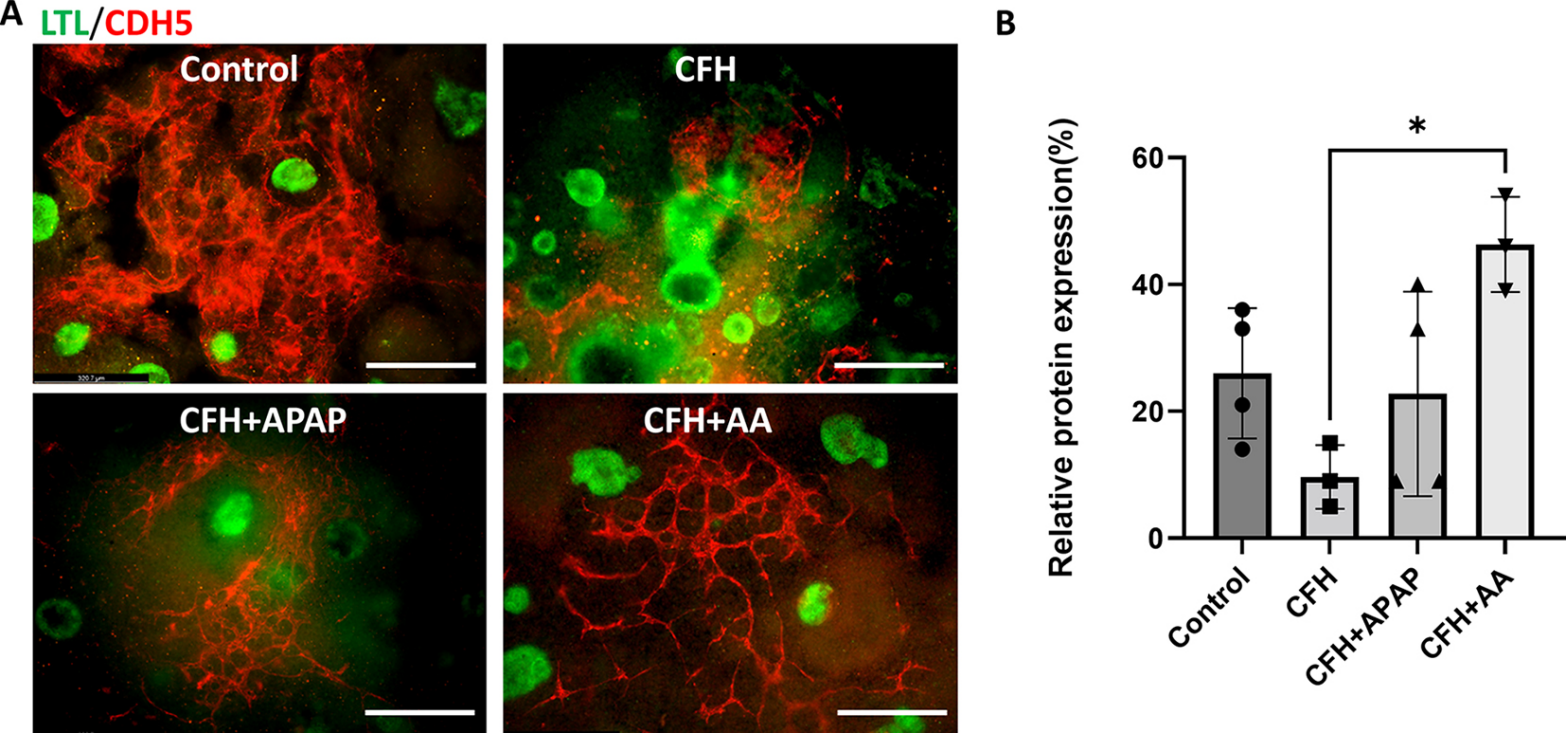
**AA improves endothelial cell marker expression in CFH-treated human kidney organoids.** (A) Immunostaining of the endothelial cell marker CDH5 (red) and the proximal tubule marker LTL (green). Scale bars: 300 μm. (B) Quantification of the images for CDH5-positive areas compared to total organoid area (DRAQ5), measured using ImageJ software (*n*>3 organoids from two independent experiments). Bars show the mean±s.d. *P<0.05 (one-way ANOVA with Tukey's multiple comparisons).

As CFH-mediated endothelial dysfunction has been reported in many organs, we evaluated staining for an endothelial cell marker in the untreated, CFH-, CFH+APAP- and CFH+AA-treated organoids. Staining for vascular endothelial cadherin (VE-cadherin or CDH5) showed an abolished vascular network in the CFH-treated organoids (Fig. 8). In contrast, kidney organoids that were co-treated with either APAP or AA maintained CDH5 staining in the presence of CFH. We found that AA treatment led to a significantly higher number of CDH5^+^ cells with better vascular networking than the APAP treatment (Fig. 8A,B).

## DISCUSSION

Increased levels of circulating CFH, which is released from red blood cells during hemolysis, contribute to sepsis-associated AKI through a variety of mechanisms including tubular obstruction, nitric oxide depletion, oxidative injury and pro-inflammatory signaling. We examined the effects of CFH in an advanced and complex *in vitro* system: kidney organoids created from human iPSCs. We discovered that CFH treatment causes tubular damage in organoids obtained by both the transwell and 3D kidney differentiation approaches (Figs 1 and 2). The results were comparable to the study by Zhang et al. (2021), which described induced apoptosis in kidney organoids treated with LPS.

Circulating CFH in sepsis can be harmful for several reasons. First, intravascular ROS (H_2_O_2_, O_2_^•−^) from activated leukocytes in sepsis can oxidize the ferrous iron (Fe^2+^) in CFH to the ferric ion (Fe^3+^, methemoglobin) and to the highly reactive ferryl radical (Fe^4+^, ferryl-CFH). Ferryl-CFH is a potent pro-oxidant that can oxidatively injure membrane lipids and other substrates (Boutaud et al., 2010). Ferryl-CFH increases over time in patients with sepsis. The heme moiety of hemoglobin can activate the macrophage inflammasome through mitochondrial ROS production (Buehler et al., 2012). CFH and Ferryl-CFH are potent oxidants and cause increased ROS in multiple cell types (Deshmukh and Trivedi, 2013; Rifkind et al., 2014; Wang et al., 2002). We discovered both elevated ROS levels and lipid peroxidation, which point to a mechanism of CFH-induced damage involving oxidative stress (Figs 1F and 2F). Image analysis indicated ROS production across multiple cell types, including tubules (Fig. 1F). This is in agreement with the previous reports that showed LPS treatment caused elevated oxidative stress in organoids (Zhang et al., 2021). Increases in cellular ROS are a major driver of mitochondrial damage because cytoplasmic ROS causes mitochondrial membrane depolarization, leading to a reduction in cellular respiration, decreased ATP production and release of cytochrome c, with subsequent activation of intrinsic apoptosis pathways (Redza-Dutordoir and Averill-Bates, 2016; Wu and Bratton, 2013). Heme-mediated organellar injury in tubule cells was first reported by Nath et al. (1998). Using a glycerol-induced model of rhabdomyolysis AKI, they reported that mitochondria and nuclei are targets of heme injury (Nath et al., 1998). Myohemoglobinuria leads to free radical production by mitochondria, which in turn causes high lipid peroxidation. Mitochondrial swelling and dysfunction can also lead to DNA damage, which has been observed mostly in the distal tubule segments of glycerol AKI models (Nath et al., 1998). Visualization of macromolecular structures by electron microscopy imaging showed loss of tubule microvilli as well as loss of endothelial cells (Fig. 3Ai,ii). The group that received CFH treatment also experienced mitochondrial swelling and enlargement (Fig. 3Aiii,Bi). After treatment with CFH, we discovered an increase in both mitochondrial segmentation and mtDNA release into the medium (Fig. 7).

Bulk RNA-sequencing analysis showed high expression of some gene transcripts, including *ROBO2*, *IGFBP5* and *TMC4* (Fig. 4A). ROBO2 is a cell adhesion molecule that forms a complex with nephrin (NPHS1) in developed kidneys. ROBO2 can negatively regulate nephrin-induced actin polymerization and it has been shown that podocyte injury can upregulate ROBO2 expression (Pisarek-Horowitz et al., 2020). IGFB5 is a secretory protein that can induce inflammation by metabolic reprogramming of glomerular endothelial cells (Song et al., 2022). IGBFP5 is upregulated in diabetic kidney disease, focal segmental glomerulosclerosis as well as chronic kidney disease (Wang et al., 2021). Together, expression of these genes suggests an unexplored effect of CFH on the cells of the glomerulus. As expected, CFH treatment increased the classic AKI injury transcript *KIM-1*. High expression of *LRP2*, *CUBN* as well as *HNF4A* was surprising as expression of these genes suggests that increased tubular differentiation occurs after injury. Analysis of the differential expression of gene pathways, including matrix metalloproteinases (MMPs) and collagens, as well as functional enrichment in epithelial to mesenchymal transition signaling and hedgehog signaling pathways, overall suggests the activation of a pro-fibrotic phenotype in response to CFH (Fig. 4B). It has been established in the literature that CFH is pro-inflammatory (Buehler et al., 2012; Irwin et al., 2015; Shaver et al., 2016). Our gene enrichment analysis similarly detected increased expression of inflammatory genes, including genes involved in TNFα signaling via NFκB and IL-6 signaling via STAT3, confirming inflammation (Fig. 4B). Interestingly, the CFH-treated group had enriched estrogen response signaling compared to control groups (Fig. 4B).

It is well recognized that systemic and intrarenal inflammation can be causal in AKI (Rabb et al., 2016). Losing the balance between pro- and anti-inflammatory mediators during AKI has a substantial impact on the degree of tissue injury (Kurts et al., 2013). Therefore, we further investigated the downstream consequences of CFH-induced kidney damage by performing cytokine array analysis. We found significant increases in angiogenin, DKK1, IL-11 and MIF (Fig. 6).

Both angiogenin mRNA transcripts and serum angiogenin protein concentrations increase in response to inflammation in humans in colorectal tissues (Etoh et al., 2000; Hartmann et al., 1999; Koutroubakis et al., 2004; Oikonomou et al., 2011). Angiogenin is produced in the renal epithelial cells of the kidney (Tavernier et al., 2017). In models of humans with AKI, the levels of angiogenin are elevated and correlate with injury (Mami et al., 2016). Increased angiogenin release was found to increase the adaptability of injured kidney tissue under endoplasmic reticulum stress, thereby attenuating the injury (Mami et al., 2016). In our model, elevated angiogenin in response to CFH likely reflects injury adaptation of tubule cells.

DKK1 is a Wnt antagonist that inhibits β-catenin-mediated WNT signaling by binding to LRP5/LRP6 with nanomolar affinity (Semënov et al., 2008). Because Wnt signaling stimulates the expression of many fibrosis-related genes in renal cells, inhibiting Wnt signaling has been shown to protect against kidney damage and reduce renal fibrotic lesions in patients with chronic kidney disease (Tan et al., 2014). DKK1 levels in the blood are elevated in a variety of disorders with infections, including viral infection, pneumonia and sepsis (Fang et al., 2014; Li et al., 2011; Mazon et al., 2018; Serrano et al., 2019). In diabetic nephropathy models, knocking down DKK1 prevented diabetes-induced renal dysfunction and microstructure deterioration (Lin et al., 2010). In our study, we observed a significant increase in DKK1 levels in organoids treated with CFH compared to those in the control group (Fig. 6Bii), suggestive of renal injury. This contrasts with the reported significant decrease in DKK1 in an organoid model of AKI treated with cisplatin, suggesting that DKK1 may have utility as a biomarker for the type of AKI (Digby et al., 2020).

IL-11 is a cytoprotective 20-kDa multifunctional member of the IL-6-type cytokine family (Goldman et al., 2001). IL-11 exerts substantial anti-apoptotic and anti-necrotic actions to protect the intestine and cardiomyocytes and to prevent endothelial cell death (Du and Williams, 1997). In mice, IL-11 reduces the inflammatory responses to LPS-induced sepsis (Trepicchio et al., 1996; Trepicchio and Dorner, 1998). The stimulation of renal tubular sphingosine kinase-1 (SPHK1) by IL-11-mediated nuclear translocation of hypoxia-inducible factor-1 (HIF-1) was protective against ischemic AKI (Lee et al., 2012). In our study, CFH treatment increased IL-11 expression (Fig. 6Biii).

MIF is a soluble immune cell-derived factor that binds to the extracellular domain of CD74 (Leng et al., 2003). Excessive inflammation and immunopathology have been associated with MIF expression, which has well-documented proliferative capabilities. CD74 is expressed on the surface of renal tubular epithelial cells (Valiño-Rivas et al., 2015). Under normal circumstances, MIF is expressed at lower levels in tubule cells and glomerular cells. The MIF-CD74 pathway plays a key role in the recovery of damaged epithelial cells by promoting cell regeneration during kidney injury (Farr et al., 2020; Shachar, 2017; Stoppe et al., 2018). MIF is a pro-inflammatory cytokine that is rapidly released into the bloodstream in various inflammatory renal diseases, including sepsis (Payen et al., 2012; Stefaniak et al., 2015; Toldi et al., 2021). We found that MIF cytokine release was significantly increased in the CFH group (Fig. 6Biv).

The long loop of Henle and distal convoluted tubules are the major sources of osteopontin in the kidney (Brown et al., 1992; Hudkins et al., 1999). Osteopontin is generated in large amounts by injured and inflamed epithelial cells in response to numerous stimuli and has anti-apoptotic effects on a number of immune cells (O'Brien et al., 1994). We observed a modest increase in osteopontin release in the CFH group compared to that in the control, but this was not significant. Future studies will further characterize of the role of the kidney in dampening inflammation in response to CFH.

Finally, hemolysis can cause endothelial cell activation. Overwhelming immune activation in sepsis can injure the endothelium, increasing the toxic effects of CFH (Opal and van der Poll, 2015). For example, circulating CFH caused endothelial dysfunction and acute lung injury in animal models of blood transfusion (Meegan et al., 2021). By stimulating endothelial cells, CFH causes upregulation of adhesion molecules through the nuclear factor κ-light-chain-enhancer of activated B-cells (NFκB) pathway and induces the release of chemokines and cytokines (Liu and Spolarics, 2003). In rodent models of sickle cell disease, high plasma levels of CFH also cause lung endothelial barrier failure and lung microvascular hyperpermeability (Ghosh et al., 2012). Hemolysis from malaria can also cause AKI, endothelial activation, and microvascular dysfunction (Barber et al., 2018). We found that CFH treatment reduced the number of endothelial cells as evidenced by lower CDH5 expression. This suggests that CFH can injure cells beyond the tubular epithelium and that further studies of the renal endothelium are needed.

To test drugs that could reverse some effects of CFH in the human kidney organoid model, we selected APAP and AA, both of which may be useful in the clinical setting (Janz et al., 2015; Kashiouris et al., 2020). Co-treating the injured organoids with AA increased the viability and decreased the expression of cleaved caspase-3 in CFH-damaged organoids (Fig. 5). Both AA and APAP may reduce the oxidative stress induced by CFH (Dunne et al., 2006). Both APAP and AA lowered oxidative stress in the injured organoids (Fig. S5). APAP prevents peroxide-driven lipid peroxidation catalyzed by hemoglobin and attenuates rhabdomyolysis-induced AKI (Boutaud et al., 2010). However, we did not see any reduction in lipid peroxidation with the co-treatment (Fig. S5).

With regard to cytokine release, the AA treatment group had lower expression of MIF and IL-11 compared to that in the control group (Fig. 6B). We found a decrease in DKK1 levels in both the co-treated groups, indicating reduced injury. AA treatment reduced osteopontin levels significantly compared to those in both the control and CFH-treated groups, suggesting that AA reduces kidney osteopontin levels in both healthy and injured conditions (Fig. 6B). Altogether, the results suggest elevated cytokine production after CFH treatment, which was mostly mitigated by AA. Both APAP and AA reduced mitochondrial segmentation, but only the AA group had a significant decrease in mtDNA released into the cell culture medium as indicated by a higher cycle threshold (Fig. 7). Finally, CDH5 expression was better maintained in the presence of AA (Fig. 8A,B).

In summary, CFH treatment reduced viability, increased cytotoxicity, and stimulated apoptosis in kidney organoids generated from human iPSCs. We discovered elevated oxidative stress, tubule cell injury, endothelial cell injury, cytokine release and ensuing mitochondrial deterioration in tubule cells. Many of the effects caused by CFH, including ROS generation, cytokine release and endothelial injury, were alleviated by co-treatment with AA. We demonstrated that the presence of CFH severely impaired human renal tubule and endothelial cell survival. However, we unexpectedly found that the kidney organoid response to CFH by secreting anti-inflammatory cytokines might mitigate further damage. Our research suggests that AA or similar molecules may minimize kidney damage in AKI in the setting of increased CFH. Additionally, future use of this human kidney organoid model of AKI will permit the dissection of the complex interplay between CFH-damaged endothelial and proximal tubule cells.

## MATERIALS AND METHODS

### Treatment of renal tubular epithelial cells with CFH

Immortalized human cortical and proximal tubular epithelial (HK-2) cells were cultured in DMEM-F-12 (Thermo Fisher Scientific, Waltham, MA, USA) with 10% FBS (wt/vol) (Life Technologies, Carlsbad, CA, USA), insulin-transferrin-selenium (ITS) solution (Corning, Corning, NY, USA), hydrocortisone (0.1 μM) and 1% penicillin/streptomycin. Cells at confluence were exposed to 1 mg/ml purified CFH for 48 h. For all experiments, we used CFH purified by high-pressure liquid chromatography from pooled blood that was collected from consenting healthy volunteers (Meegan et al., 2021). All clinical investigation was conducted according to the principles expressed in the Declaration of Helsinki. The study was conducted under IRB #061088 ‘Biomarkers in Normal Plasma’ approved by Vanderbilt University Medical Center's Institutional Review Board. After 48 h, the control or treated cells were harvested for immunostaining or assays.

### Kidney organoids from iPSCs

Kidney organoids were generated from iPSCs (American Type Culture Collection, DYR0100; origin, SCRC-1041 foreskin fibroblast cell line) using an established protocol (Przepiorski et al., 2018; Takasato et al., 2015). Briefly, iPSCs were grown on Geltrex-coated (Thermo Fisher Scientific) plates in mTesR medium (STEMCELL Technologies, Cambridge, MA, USA) medium. After reaching confluency, the iPSCs were dissociated with Accutase (Thermo Fisher Scientific), seeded onto Geltrex-coated plates at a density of 100,000 cells/cm^2^ and treated with mTesR medium with 10 μM ROCK pathway inhibitor Y-27632 (dihydrochloride) (STEMCELL Technologies) for the first 24 h. iPSCs were treated with mTesR media without ROCKi for the next 48 h. From day 3, iPSCs were treated with APEL medium (STEMCELL Technologies) with 2% Protein Free Hybridoma Medium II (PFHMII; Thermo Fisher Scientific) containing 8 μM of the Wnt activator CHIR99021 (CHIR, Reagents Direct, Encinitas, CA, USA) for the first 5 days. The intermediate mesoderm cells derived were then treated with 200 ng/ml FGF9 (Peprotech, Cranbury, NJ, USA) and 1 µg/ml heparin (Sigma-Aldrich, St. Louis, MO, USA) for two more days for differentiation into nephron progenitors. On day 7, the cells were dissociated and grown on transwells with a 1-h pulse of 5 μM CHIR followed with FGF9+heparin treatment until day 12. From day 12 onwards, the organoids were treated with APEL+PFHMII containing heparin. The differentiated organoids were harvested between days 21 and 25 for further experiments or analysis. Alternatively, to derive 3D suspension kidney organoids, another established protocol was used (Digby et al., 2020). iPSCs were dissociated and grown as embryoic bodies in the presence of 8 μM CHIR for the first 3 days. The embryoic bodies were then treated with DMEM (Thermo Fisher Scientific) containing Knockout Serum Replacement™ (Thermo Fisher Scientific) for the next 23 days to derive the 3D kidney organoids. The area of organoids positive for nephron segment markers such as CDH1 for whole tubules and LTL for proximal tubule was used to measure the efficiency of differentiation. The efficiency of differentiation was quantified by ImageJ analysis by comparing the area positive for these markers to the total area of the organoids as measured by DAPI staining.

### CFH cytotoxicity in organoids

Kidney organoids were treated with purified ferrous CFH isolated from normal human blood at a concentration of 1 mg/ml for 48 h to induce toxicity (Pishchany et al., 2013). The viability and cytotoxicity of the organoids treated were analyzed by the CyQUANT™ LDH Cytotoxicity Assay Kit (Thermo Fisher Scientific) and MTT assay (Sigma-Aldrich). To evaluate the impact of small molecules that can function as hemoprotein reductants, the organoids were co-treated with CFH and clinically relevant concentrations of APAP (1000 nM, Sigma-Aldrich, A7085) or AA (200 nM, Thermo Fisher Scientific, A61-100) for 48 h. APAP was solubilized in ethanol whereas AA was dissolved in water.

### Immunocytochemistry

Organoids were fixed using 4% paraformaldehyde (Thermo Fisher Scientific) for 30 min. The organoids were then washed with PBS (Thermo Fisher Scientific) and blocked with 10% donkey serum (Millipore Sigma) in PBS containing 0.03% Triton X-100 (Sigma-Aldrich) for 30 min. The organoids were washed with PBS and stained with primary antibodies (Table S1) overnight at 4°C. The next day, the organoids were washed with PBS and stained with corresponding secondary antibodies (Table S1). Hoechst 33342/DAPI or DRAQ5 (65-0880-92, eBioscience, Thermo Fisher Scientific) was used for nuclear staining. Fluorescently stained organoids were then imaged using a confocal microscope (Zeiss LSM710/Nikon spinning disk). Fluorescence images were analyzed using ImageJ software.

### Biochemical assays

#### MTT assay

The organoids were washed with PBS and then treated with MTT reagent (Sigma-Aldrich; 1 mg/ml) for at least 60 min, leading to the purple formazan formation in living cells. The reagents were then removed and isopropanol was added to dissolve the insoluble formazan. The absorbance of the colored solution was measured using a microplate reader at 560 nm and 690 nm. Background absorbance at 690 nm was subtracted from absorbance at 560 nm and the optical density (OD) values were plotted to measure cell viability.

#### LDH assay

LDH release from the cells was evaluated using the CyQUANT™ LDH Cytotoxicity Assay Kit. Briefly, the cell culture medium was collected for other purposes at 48 h after treatment. The cells were treated with 10% Triton X-100 and incubated for 15 min for maximum LDH release from the sample. The cell culture medium was collected and 50 µl of each sample was transferred into a 96-well opaque-walled, non-transparent assay plate. The LDH detection reagent was prepared by combining the LDH detection enzyme mix and reductase substrate. Then, 50 µl of detection reagent was added to each well. The wells were incubated for 60 min at room temperature. Luminescence was recorded on a plate reader at 490 nm and the corresponding OD was plotted to quantify cell death.

#### ROS assay

Deep Red CellROX (Thermo Fisher Scientific) reagent was added to the culture medium at a final concentration of 5 μM and incubated for 30 min at 37°C. The medium was then removed and cells were washed three times with PBS. At this stage, the cells were fixed using 4% paraformaldehyde and DAPI (1:1000) was added for nuclear staining. The cells were then washed three times with PBS, transferred to coverslips and imaged using a Nikon spinning-disk fluorescence microscope.

#### ELISA

The concentration of KIM-1 in the cell culture supernatant was determined using a Human Serum TIM-1/KIM-1/HAVCR Quantikine ELISA Kit (R&D Systems, Minneapolis, MN, USA) following the manufacturer's protocol. Briefly, the cell culture medium was diluted using the calibrator diluent in the kit (1:4) and then added to microplates pre-coated with human KIM-1. Treatment with a series of solutions/washes resulted in a yellow product. Absorbance was measured using a plate reader at 495 nm and compared to that of the Quantikine Kit standards.

#### MDA assay

Free radicals present in cell culture can increase peroxidation of lipids present on cell membranes, resulting in cell damage. Lipid peroxidation causes formation of reactive aldehydes including MDA, which can be used to assess oxidative stress. The MDA assay was carried out using a commercially available kit (ab118970, Abcam) following the manufacturer's instructions. Briefly, 200 µl of cell culture supernatant was aliquoted for the assay. MDA standards were also prepared. Then 600 µl of the thiobarbituric acid reagent was added both to the samples and to the MDA standards and incubated at 95°C for 60 min. Tubes were cooled to room temperature using an ice bath for 10 min, 200 µl of the cooled solution was added to one well of 96-well plates (three biological replicates), and the absorbance was immediately measured on a microplate reader to calculate the OD at 532 nm. The standard curve was plotted, and the MDA concentrations of the samples were calculated. Increased MDA concentration is associated with increased oxidative stress.

#### SOD assay

The SOD assay was carried out using the commercially available SOD kit (Abcam, Waltham, MA, USA) following the manufacturer's instructions. The cell culture medium was collected and 20 µl of each sample was used for analysis. Samples were prepared with and without addition of SOD enzyme solution for the blank correction. To the 20 µl sample, 200 µl of the WST working solution was added. The assay uses a tetrazolium salt WST-1, which produces a water-soluble formazan dye upon reduction with superoxide anion. The WST-1 reduction is linearly related to SOD-mediated inhibition activity of xanthine oxidase. SOD catalyzes the dismutation of the superoxide anion into hydrogen peroxide and molecular oxygen, resulting in decrease of WST-1 reduction. The inhibition activity of SOD was measured by colorimetric measurement of OD at 450 nm.

### Human cytokine array

The human cytokine proteome profiler array is a membrane-based sandwich immunoassay that can identify 102 cytokines. Conditioned media from the cultured organoids with or without treatments were aliquoted and samples were stored at or below −20°C. The samples were thawed and analyzed using the Proteome Profiler Human XL Cytokine Array (R&D Systems). The membrane was blocked for 1 h on a rocking platform shaker, then incubated with samples overnight at 2-8°C. The membranes were then washed with wash buffer for 10 min on a rocking platform shaker. The detection antibody cocktail was added, and samples were incubated for 1 h on a rocking platform shaker. Next, 1× streptavidin-HRP was added and incubated for 30 min at room temperature. Finally, the Chemi reagent mix was added and chemiluminescence signals were obtained using a Bio-Rad ChemiDoc MP Imaging system. The intensities of signals were quantified using ImageJ software.

### TEM imaging

Organoids were fixed in a mix of 2.5% glutaraldehyde and 1% paraformaldehyde and stored at 4°C. After primary fixation, the organoids were sequentially post-fixed in 1% tannic acid, 1% OsO_4_, and *en bloc* stained in 1% uranyl acetate. The samples were subsequently dehydrated in a graded ethanol series and infiltrated with Quetol 651-based Spurr's resin (Electron Microscopy Sciences) using propylene oxide as the transition solvent. The resin was polymerized at 60°C for 48 h. All samples were cut on a Leica UC7 ultramicrotome with a nominal thickness of 70 nm and collected onto 300 mesh nickel grids. TEM was performed on a Tecnai T12 transmission electron microscope operating at 100 keV using an AMT NanoSprint5 CMOS camera. Endothelial and tubular structures were tiled using SerialEM automation software and reconstructed using the Etomo software suite (Kremer et al., 1996).

### RT-PCR

Total DNA was isolated from cell culture supernatant using the DNeasy Tissue Kit (Qiagen, Germantown, MD, USA) according to the manufacturer's protocol. The RT-PCR reaction was carried out using 20× PrimePCR Assay or PrimePCR Control Assay. Quantitative RT-PCR reactions were performed on a Bio-Rad CFX system (Hercules, CA, USA), using SYBR1 Green PCR Master Mix (Applied Biosystems, Waltham, MA, USA). The amplification reactions were performed as follows: 2 min at 95°C, 40 cycles of 95°C for 5 s and 60°C for 30 s, and 65-95°C (0.5°C gradient) for 5 s per step. C_q_ values were plotted to estimate the DNA content for mitochondrially encoded cytochrome c oxidase III (*MT-CO3*) and mitochondrially encoded NADH ubiquinone oxidoreductase core subunit 4L (*MT-ND4L*).

### Bulk RNA sequencing

Using the Qiagen RNAEasy kit, total RNA was generated from triplicate samples of control and CFH-treated organoids. The Vanderbilt Technologies for Advanced Genomics (VANTAGE) core carried out library preparation and sequencing on an Illumina NextSeq 500 sequencer. We used Basepair next-generation sequencing data analysis software for the analysis. Differential expression analysis was carried out with a *P*<0.05 (−log_10_*P*=1.3) and a threshold of log_2_FC=2 (Digby et al., 2020). Additional analysis was performed by Vanderbilt Technologies for Advanced Genomics Analysis and Research Design (VANGARD). Briefly, the reads were trimmed by cutadapt (https://cutadapt.readthedocs.io/en/stable/) and were mapped to the human genome. The differential expression analysis was carried by DESeq2 (v1.30.1) (https://bioconductor.org/packages/release/bioc/html/DESeq2.html). The RNAs with absolute fold change≥2 and false-discovery rate adjusted *P*-value≤0.05 were detected as significantly differentially expressed.

### Quantification and statistics

Statistical significance for all data was determined using one-way ANOVA or unpaired two-tailed *t*-test in Prism (GraphPad) with Tukey's multiple comparison test for post hoc analysis. *P*≤0.05 was considered to be statistically significant.

### Study approval

This study contains no human subjects or animals.

## Supplementary Material

10.1242/dmm.050342_sup1 Supplementary information

Table S2. CFH Vs Control-DESeq2 data
