# Supplementary material for: Ascorbate protects human kidney organoids from damage induced by cell-free hemoglobin
Source: Dis Model Mech. 2023 Dec 1;16(12):dmm050342. doi: 10.1242/dmm.050342 (PMC10695115; doi:10.1242/dmm.050342)
Supplement: Supplementary information [file dmm-16-050342-s1.pdf]

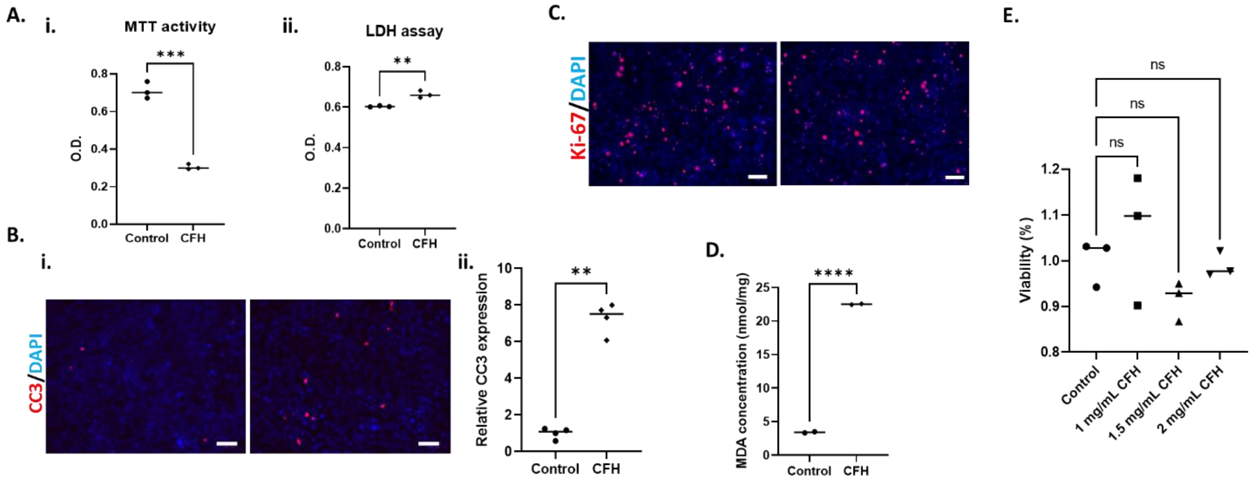

Fig. S1. CFH increased apoptosis in HK-2 renal tubular epithelial cells. (A) CFH treatment reduced MTT activity (i) and increased LDH activity (ii) in HK-2 cells (n=3). (B) Cleaved caspase-3 (CC3) staining (i) showing increased expression within the CFH treated group and corresponding quantification (ii) (C) Ki-67 staining of the CFH treated group showing no significant difference from the control group. (D) CFH-treated cells showing lipid peroxidation by MDA assay. Scale bar 100 µm. (E) MTT assay showing batch variability in HK2 cells. **p <0.01,*** p <0.001, **** p ≤0.0001

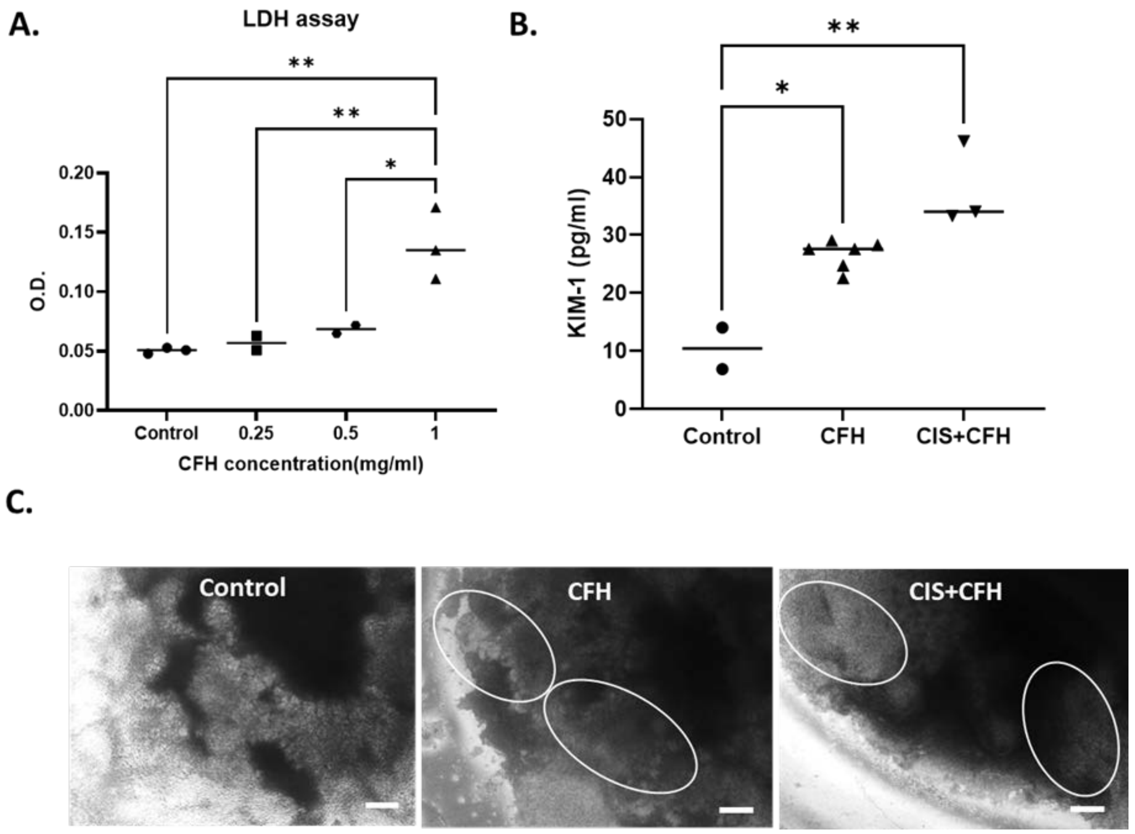

Fig. S2. Cisplatin (CIS) co-treatment with CFH increases injury in kidney organoids. (A) LDH activity (O.D.) in organoids treated with different concentrations of CFH identified 1 mg/mL as a sufficient dosage to induce injury. (B) KIM-1 ELISA showing increased KIM-1 release from the CIS+CFH treated organoids than the CFH alone organoids into the tissue culture media. (C) The brightfield images of the whole organoids treated with both CFH and CIS + CFH losing differentiated tubule segments (white ovals). Scale bar 50 µm. n=3, *p <0.01,** p <0.01.

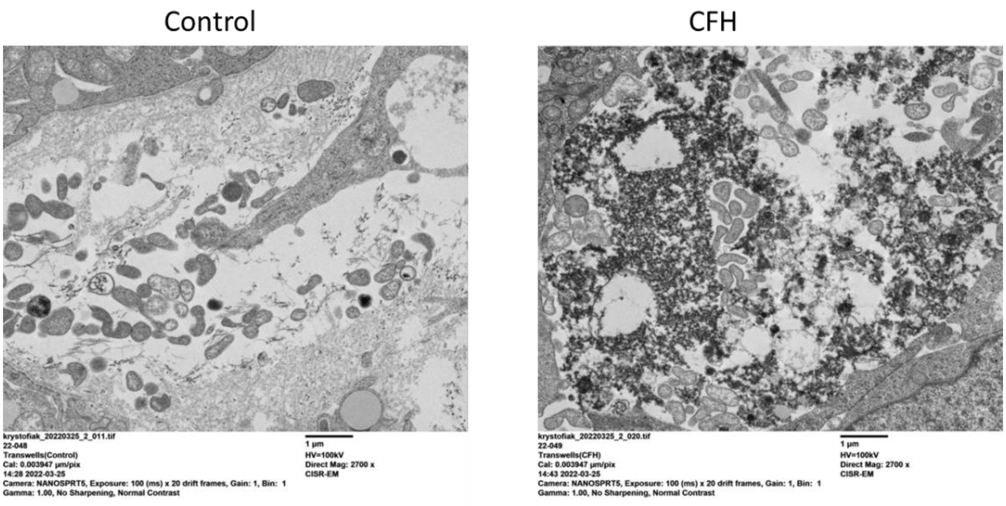

Fig. S3. The kidney organoids treated with cell-free hemoglobin have apoptotic cells. (A) Representative transmission electron microscopic images of apoptotic cells in human kidney organoids either untreated or treated with CFH. Scale bar, 1 µM.

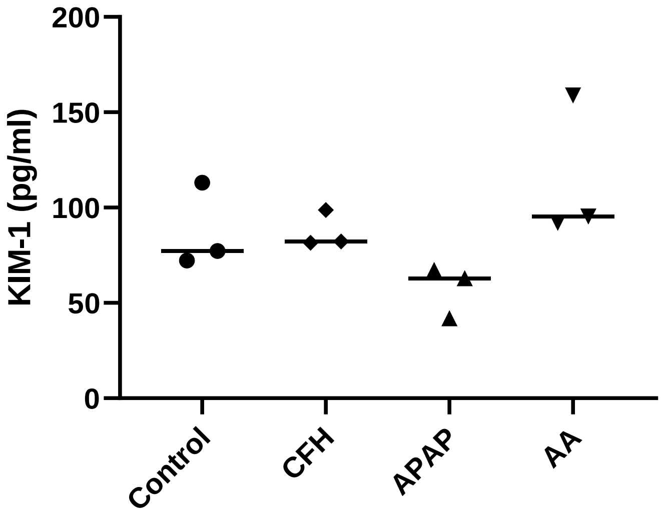

Fig. S4. APAP co- treatment shows modest decrease in KIM-1 release in CFH organoids. KIM-1 release by ELISA analysis of organoid media from the four different groups of organoids including the control, CFH-treated, CFH+APAP treated and CFH +AA treated organoids.

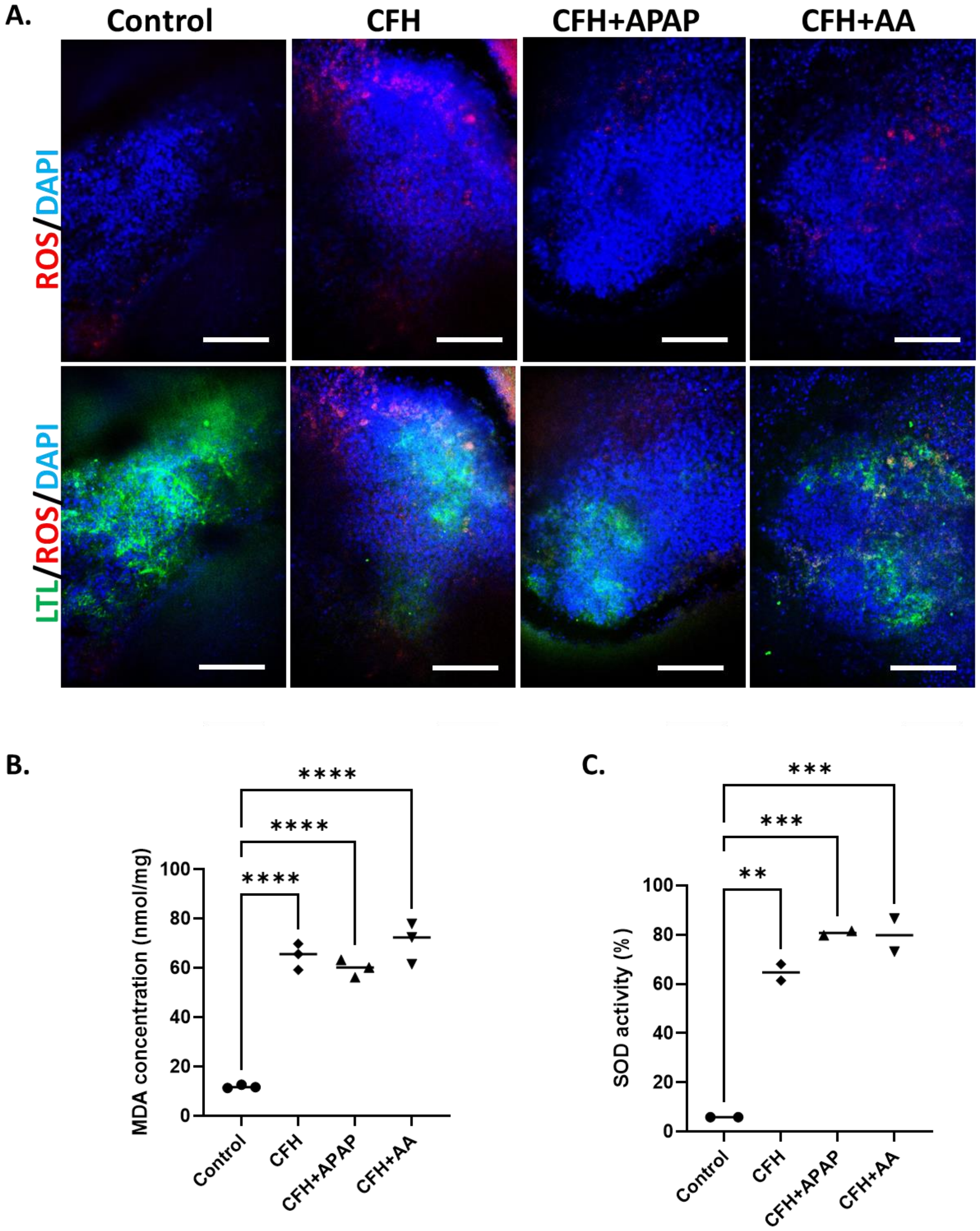
A.
Control
CFH
CFH+APAP
CFH+AA
ROS/DAPI
LTL/ROS/DAPI
B.
MDA concentration (nmol/mg)
100
80
60
40
20
0
****
****
****
Control
CFH
CFH+APAP
CFH+AA
C.
SOD activity (%)
100
80
60
40
20
0
***
***
**
Control
CFH
CFH+APAP
CFH+AA

Fig. S5. Acetaminophen and ascorbate reduced ROS induced by CFH. The experiments to analyze reactive oxygen species (ROS) and downstream effects were performed on untreated human kidney organoids (control), organoids treated with cell-free hemoglobin (CFH), CFH and acetaminophen (CFH+APAP), or CFH and ascorbic acid (CFH+AA) for 48 h. (A) ROS assay using Deep Red ROS reagent (red) co-stained with tubule marker LTL (green). DAPI stain the nuclei (blue). Scale bar is 100 µm. (B) Lipid peroxidation analysis using MDA assay. (C) Superoxide dismutase activity as measured by SOD assay. *p <0.05, **p <0.01.

Disease Models & Mechanisms • Supplementary information

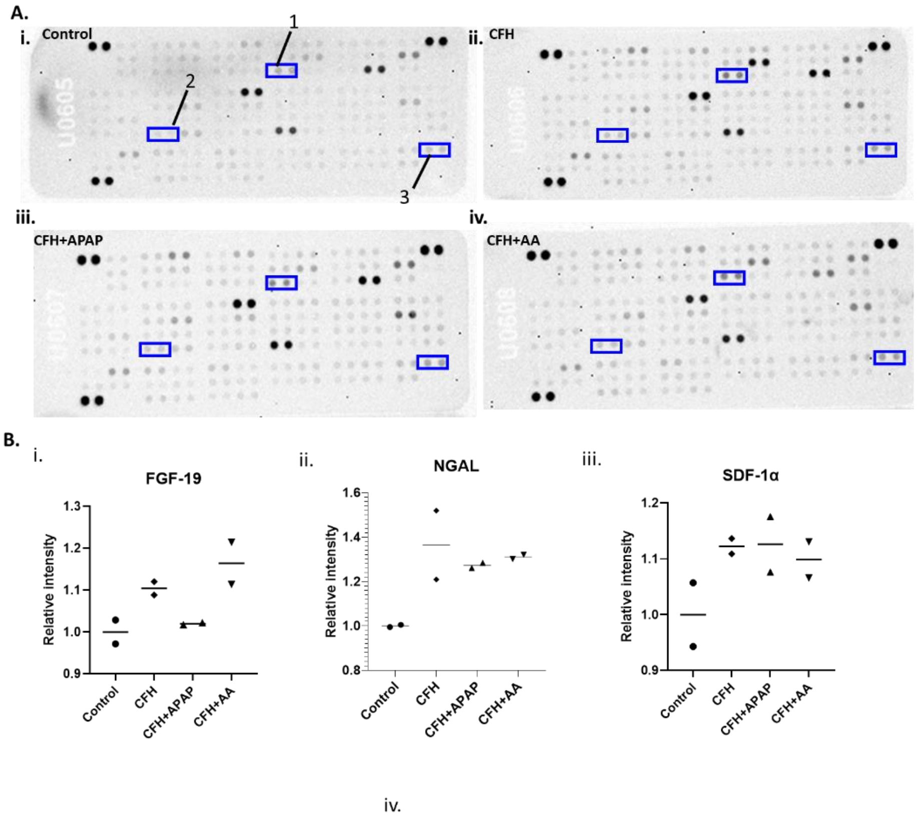

1. FGF-19
2. NGAL
3. SDF-1α

C.

Cytokine	CFH to Control	CFH+APAP to Control	CFH+AA to Control
DKK1	***(0.0003)	**(0.0053)	**(0.0020)
IL-11	*(0.0287)	*(0.0299)	ns(0.5344)
MIF	**(0.0088)	**(0.0047)	ns (0.1874)
Osteopontin	ns (0.4337)	ns (0.6267)	ns (0.9896)
Angiogenin	* (0.0427)	ns(0.0683)	ns(0.0766)
FGF-19	ns (0.2078)	ns (0.9635)	ns (0.0591)
NGAL	ns(0.0947)	ns(0.2044)	ns(0.1467)
SDF-1α	ns (0.2982)	ns(0.2821)	ns(0.4414)

Fig. S6. Cytokine array analysis. (A) Cytokine array analysis of culture media collected from dishes containing human kidney organoids from the following 48 h treatment groups: (i) untreated control, (ii) cell-free hemoglobin (CFH), (iii) CFH and acetaminophen (CFH+APAP), and (iv) CFH and ascorbic acid (CFH+AA). Factors that appeared by eye to be increased in the CFH group compared to untreated control are marked in the blue boxes. (B) Quantification of cytokine levels in ImageJ software for: (i) FGF-19, (ii) Lipocalin 2 (NGAL), and (iii) C-X-C Motif Chemokine Ligand 12 (SDF-1α). (C) Table showing the p-values for the different cytokines in all treated groups as compared to control group.

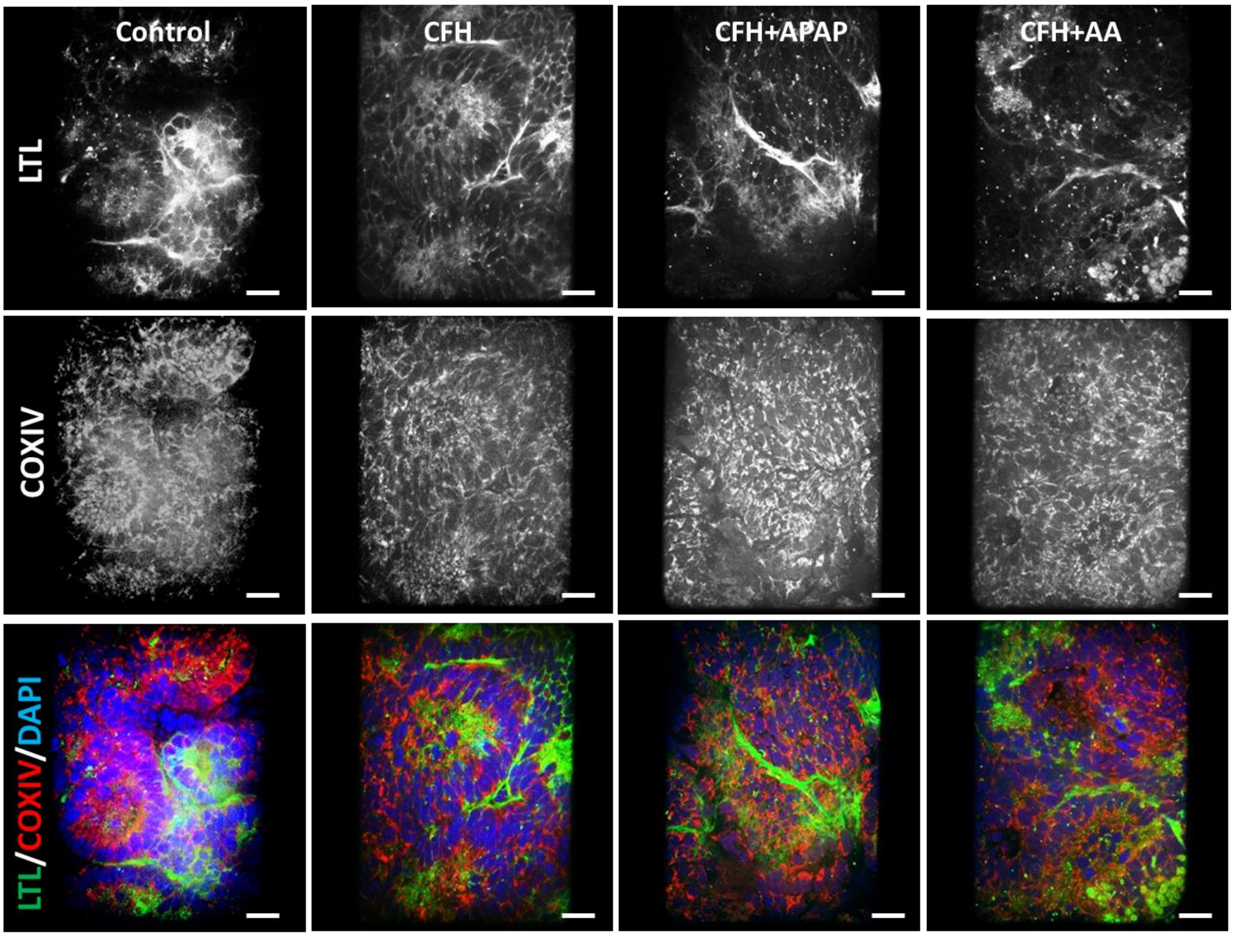

Fig. S7. Acetaminophen or ascorbic acid did not alter proximal tubule cell marker expression in the cell-free hemoglobin-treated human kidney organoids. (A) Immunostaining with mitochondrial marker COXIV (red) and proximal tubule marker (LTL) (green) co-stained with DRAQ5 to mark the nuclei (blue) showing no significant difference in the expression of the proximal tubular epithelial marker LTL in the CFH-treated and co-treated transwell organoids. Scale bar 20µm.

Table S1. List of antibodies

Primary Antibodies						
	**Target**	**Origin**	**Isotype**	**Company**	**Catalog#**	**Dilution**
Distal/Proximal tubule	ECAD/CDH1	Mouse	IgG	Fisher Scientific	BDB610181	1:200
Collecting duct	GATA3	Goat	IgG	Fisher Scientific	AF2605-SP	1:200
Podocytes	PODXL	Rabbit	IgG	Proteintech	18150-1-AP	1:200
Mitochondria	COXIV	Mouse	IgG1	Cell Signaling Technology	11967	1:200
Endothelial cells	VE-Cadherin/CDH5	Rabbit	IgG	Cell Signaling Technology	93467	1:200
Proliferation	Ki-67	Rabbit	IgG	Abcam	ab16667	1:200
Apoptosis	Cleaved Caspase 3	Rabbit		Cell Signaling Technology	11967	1:200
Visualization	Binds to	Stain	Channel	Company	Catalog#	Dilution
Nuclei	DNA	DAPI	blue	Millipore Sigma	D9542	1:1000
Nuclei	DNA	DRAQ5	far-red	Thermo Fisher Scientific	65-0880-92	1:1000
Lectin						
Proximal tubule	Fucose	Lotus Tetragonolobus Lectin (LTL)	Green	Vector Laboratories	FL-1321-2	1:200

Secondary antibodies					
**Species**	**Fluorophore**	**Target**	**Company**	**Catalog#**	**Dilution**
Goat	Alexa 488	Anti-mouse IgG	Life technologies	A-21131	1:200
Goat	Alexa 594	Anti-mouse IgG	Thermo Fisher Scientific	A-11032	1:200
Donkey	Alexa 594	Anti-goat IgG	Thermo Fisher Scientific	A-11055	1:200
Donkey	Alexa 594	Anti-rabbit IgG	Thermo Fisher Scientific	A-21207	1:200

Table S2. CFH Vs Control-DESeq2 data

Available for download at
https://journals.biologists.com/dmm/article-lookup/doi/10.1242/dmm.050342#supplementary-data
